# “I Had to Do It All Alone”: Hispanic Perspectives on Navigating Breast Cancer Treatment

**DOI:** 10.3390/ijerph20054163

**Published:** 2023-02-25

**Authors:** Eunjeong Ko, France Nguyen-Grozavu, Andrea Valadez Galindo

**Affiliations:** School of Social Work, San Diego State University, San Diego, CA 92182, USA

**Keywords:** COVID-19, barriers, challenges, Hispanic, breast cancer patients

## Abstract

Cancer patients are at a high risk for COVID infection and its corresponding impacts on treatment delay, social isolation, and psychological distress. Hispanic breast cancer patients may be more vulnerable due to a lack of resources and language barriers, widening disparities in cancer care. This qualitative study explored the challenges and obstacles to cancer care during the COVID pandemic among 27 Hispanic females from a United States–Mexico border region. Data were collected via individual in-depth interviews and analyzed using thematic analysis. The majority of the participants were interviewed in Spanish. More than half (55.6%, n = 15) were diagnosed with breast cancer within the prior year to the interview. One-third of the participants (33.3%, n = 9) reported that COVID somewhat to greatly impacted their cancer care. Study findings revealed potential barriers and challenges for cancer care at multiple levels (e.g., medical, psychosocial, financial level) during the COVID pandemic. Five major themes reported include: (1) delays in testing and access to care; (2) fear of COVID infection; (3) social isolation and reduced social support; (4) challenges in navigating treatments alone; and (5) financial hardships. Our findings highlight the importance for health care practitioners to understand various challenges encountered by underserved Hispanic breast cancer patients due to COVID. Screening for psychological distress and exploring approaches to expand social support to address these challenges are discussed.

## 1. Introduction

The ongoing COVID-19 pandemic has been a major threat to public safety and has resulted in unprecedented psychological distress and illness among individuals across the world [1,2,3]. Individuals diagnosed with cancer are particularly vulnerable because of their compromised immune status, which increases the risk for COVID infection and disruption in cancer care treatments and delivery [3,4]. Increased susceptibility to COVID infection and its adverse effects, such as hospitalization and mortality, has caused significant psychological distress among cancer patients. 

A recent study with breast cancer (BC) patients found that half of the participants had a fear of COVID impacting their cancer care or recovery, and 66% of the participants had anxiety about contracting COVID [5]. In addition, mitigating measures for COVID (e.g., social distancing, isolation, and lockdown) reduced social support, imposed a sense of social isolation, and increased psychological distress among cancer patients [6,7]. For example, limited social contact and a visitor ban at hospitals and health clinics added to cancer patients’ vulnerability, especially during a time when they are most vulnerable and in need of support [6]. 

After skin cancer, BC is the second most common cancer among women [8]. In particular, BC is the most commonly diagnosed and is the leading cause of death among Hispanic females [9]. Cancer disparities have been persistent during the COVID pandemic, with racial/ethnic minorities having a disproportionately higher mortality and lower access to cancer care [10,11]. A recent review reported that racial/ethnic minorities such as African American and Hispanic BC patients experienced greater morbidity and mortality rates due to COVID-19 when compared to non-Hispanic Whites [11]. In addition, Hispanic BC patients had a greater likelihood of having involuntary treatment delays, food insecurity, and concerns for financial security throughout the COVID pandemic compared to their White counterparts [12]. Hispanic cancer patients also experience a lack of insurance coverage, language barriers, and limited health literacy [13,14]. 

Cancer patients who live near the United States (US)–Mexico border region may face additional challenges, such as complex coordination of care, physical distance to treatment facilities, and health insurance comparability between the two nations [13,14]. It is common for Hispanic patients to travel to border towns both in the US and Mexico when accessing health care services [15]. Changes in regulations and policies for border crossing during the COVID pandemic may further complicate existing challenges for care coordination [16]. 

Although there are some studies that discuss cancer patients’ experiences and concerns during the COVID pandemic and patient needs in general [6,7,17,18,19], there remains a dearth of literature exploring the extent of COVID-related challenges and their implications for accessing cancer care among Hispanic BC patients from border areas. Lockdown policies have been lifted and social distancing policies have been removed, yet BC patients continue to be affected by the COVID pandemic, thus impacting their health care access and utilization. This study aimed to explore the experiences of Hispanic BC patients related to their barriers and challenges in navigating cancer care during the COVID pandemic. 

## 2. Materials and Methods

### 2.1. Study Design and Setting

This is a qualitative study using in-depth interviews with Hispanics with BC in a border city in San Diego, California. San Diego is located on the US–Mexico border across from the city of Tijuana, Baja California, Mexico. The region is home to a vibrant binational population that crosses the border daily, both north- and southbound, for work, shopping, commerce, family, and health care services [15]. A local non-profit cancer organization that provides psychosocial and case management support (e.g., financial navigation) for cancer patients assisted with recruitment of participants. 

### 2.2. Sampling Method and Participants

Convenience and purposive sampling methods were used to recruit potential participants. Participants’ eligibility criteria were self-identified Hispanic female, aged 18 years and older, diagnosis of BC, and cognitive competence. A staff member from the local organization screened and provided to potential participants a recruitment flyer approved by the San Diego State University Institutional Review Board (IRB). Those who expressed interest in participating provided their contact information to the research assistant and were scheduled for an interview. Among the 53 potential participants, five participants could not be reached and five were later deemed ineligible. Of the 43 eligible participants who initially agreed to participate in the study, eight participants later refused for various reasons (i.e., feeling ill, discomfort) and another eight failed to show up for the interviews. Therefore, a total of 27 women were interviewed in this study. 

### 2.3. Data Collection

Face-to-face interviews were conducted from March 2021 to June 2021 via Zoom or phone call by a trained bilingual graduate research assistant using a semi-structured interview guide. This data collection method was optimal due to the “stay-at-home” order issued in California and the US–Mexico border closure during the study period. Interviews were conducted either in English or Spanish and lasted approximately 40 minutes to 1 hour. Interview guide questions included: (1) How has COVID-19 impacted your cancer care? (2) How has COVID-19 impacted the availability of the support you receive from your family, friends, and community? and (3) What has contributed to your distress, if any? 

The research assistant also took field notes during the interviews, such as description of the setting, participants’ nonverbal and other pertinent behaviors, and the research assistant’s reflections about her own performance throughout the interview process. These additional notes provided important context to inform the interpretation of the data [20]. The study procedure was reviewed and approved by the San Diego State University IRB (HS-2021-0049).

### 2.4. Data Analysis 

In-depth interviews were audio-recorded, transcribed, and translated from Spanish to English by a second research assistant. The two research assistants conferred and discussed issues to ensure clarity and improve the quality of the translated data. Data analyses were based on transcripts from the recorded interviews, and the transcripts were reviewed multiple times. Initial analyses of the transcripts included manual coding of the data, which was performed independently by two researchers (EK and FN). Each coder separately identified emergent themes and salient patterns after the initial coding of the data, using a thematic analysis approach [21]. The qualitative data analysis included combining themes that were similar and rephrasing categories that differed between the coders to improve clarity and trustworthiness of data interpretation. Each researcher independently read the coded transcripts and then engaged in continuous joint discussions to clarify coding differences and achieve consensus in the development of a final list of thematic categories. Selective coding was conducted when determining quotes that best illustrated the perceived barriers and challenges of navigating care by Hispanic BC patients during the COVID pandemic. 

## 3. Results

### 3.1. Participants’ Sociodemographic Characteristics and Cancer-Related Information 

The participants’ characteristics and cancer-related information are reported in Table 1. The participants’ average age was approximately 54 years, and the majority (88.9%, n = 24) were interviewed in Spanish. More than half (55.6%, n = 15) were diagnosed with BC within a year prior to the interview, and about 41% (n = 11) were diagnosed at stage 2. While the majority (63.0%, n = 17) reported no COVID impacts on their cancer care, nine participants (33.3%) reported that their cancer care was somewhat or extremely impacted by COVID. Qualitative analysis yielded five major themes relating to medical, psychosocial, and financial challenges. 

### 3.2. Delays in Testing and Access to Care 

Most participants reported no COVID-related impact on cancer treatments (e.g., radiation, chemotherapy). However, some participants shared the difficulty of making appointments for diagnostic testing (e.g., biopsy). A participant shared her frustration for delays in making a diagnostic appointment.

*
When I was wanting to make the appointment for the biopsy, they scheduled it until a month later instead of expediting it. They made me wait a month and that stressed me so much because I wanted to get all the testing done quickly.* (P10)

Another participant also recalled the challenges in making a clinic appointment. Inability or difficulties scheduling necessary appointments caused much anxiety.

*
Obviously, setting up an appointment was complicated because as soon as clinics started opening up appointments, they would fill up really fast. So everything kept getting pushed back…Well, we have always thought that cancer is equivalent to death. What has always worried is not so much the illness but the ability to have access to the cancer care. That is what truly kills you. Because you are distressed, you are thinking, you are worried all the time...* (P9)

### 3.3. Fear of COVID Infection 

Participants expressed heightened fear of potential increased risk to contract COVID-19, especially since the virus is airborne. Despite the COVID regulations and precautionary measures, participants noticed the risk of COVID exposure in the clinic and felt uneasy about it.

*
I sometimes feel uncomfortable because other people are coughing. I do worry for my health…I do notice that sometimes the workers touch things that other patients already touched, or they sometimes don’t change gloves. It worries me because of COVID.* (P6)

Participants who were previously infected with COVID were worried about the potential serious effects compounding their already immuno-compromised status. Some participants had reported personal COVID morbidity and mortality experiences about their family members, relatives, and friends. A participant shared her experience about potential mortality during her quarantine with the COVID infection.

*
The day I knew I had COVID I stayed home completely by myself. I think that was the saddest part of this experience. What if I got really sick or something bad happens and there is no one around. To top it off, I started hearing of loved ones who passed away from COVID. To me, it was shocking and alarming to have been exposed to COVID because I am currently a cancer patient and I thought I would probably die...to be honest, I did not want to fall asleep because I was scared that I would not wake up.* (P46)

There were also widespread concerns about COVID infection potentially interrupting treatment routine and completion.

*
I was very worried because I felt that due to the treatment, my immune system was more compromised. I was one of the vulnerable people and yeah I got worried because I said oh my god, I thought that I would not be able to see the doctor.* (P13)

### 3.4. Reduced Social Support and Social Isolation 

Due to the border closure, participants who were from and had family in Mexico experienced a great sense of distress due to the physical separation from family members. They found adjusting to the new environment more challenging without their family members nearby.

*
Right now, they are not able to cross (the border) because of the COVID border restrictions. I haven’t been able to go out or spend time with people. Not being able to see my loved ones has affected me a lot…Well everything reminds me of home, I do sometimes feel lonely.* (P3)

A sense of social isolation during the COVID lockdown was pervasive among the participants. Most perceived social distancing as a necessary measure for safeguarding from COVID exposure, yet it was challenging as it prevented the participants from physically interacting with loved ones. Limited physical contacts with family left them emotionally vulnerable, particularly during cancer treatments when they would need support the most.

*
After the treatments and everything that I went through, nobody would come to my house. They would only call me on the phone. No physical contact, you know.* (P10)

*
Being away from my family is difficult for me. At least I can talk to them on the phone and make video calls, but it is still not the same as having them physically with me. You can’t cry and have them comfort you.* (P1)

Some participants continued participating in support programs in a virtual setting. However, it was considered less personal, and it limited social interactions, thus impeding networking and the formation of relationships with others who shared a similar health situation.

*
The support groups were no longer available because of COVID and those really helped me during those difficult times. There have been zoom meetings but it is not the same as going in person…Being in person makes it so much better. When the meetings are virtual, only 3 people go and it is the ones who already went through cancer, it is not the ones who have been recently diagnosed.* (P13)

### 3.5. Challenges in Navigating Treatment Alone

Participants expressed a great deal of anxiety and concerns regarding going to the clinic and navigating care and treatment by themselves without their family members. For instance, receipt of bad news during cancer treatment was difficult to bear in the absence of family support and increased emotional vulnerability when having to attend appointments alone.

*
I’ve been hospitalized, and I’ve had to go through it alone…It is impacting because you are being put under anesthesia, and you enter surgery and you do not know what is going to happen. And during appointments, you might be told bad news. Of course, I am listening and talking, but at times the emotions are too strong. It is not the same thing when they (family) are with you in the hospital waiting for you to wake up from surgery and on track of what comes next.* (P9)

The absence of having family present to accompany them during clinic visits due to the visitor ban heightened linguistic barriers and difficulties in communicating with clinicians, which can exacerbate the concern about a lack of understanding essential information given by the clinician. Family members may help translate and serve as an interpreter during clinic visits, so their absence evoked fear and anxiety of missing important information.

*
Once the pandemic started, no one was allowed to go in with me. I felt confident with the doctors but since I don’t know English, I would feel a little nervous and embarrassed. My children know English so the two times that they were able to accompany me, they would translate. After that, I was scared at first that I would not truly comprehend any important information that was given to me…* (P46)

### 3.6. Financial Hardship 

Financial hardship following receipt of a BC diagnosis was identified as a significant source of distress. Disruption in employment was a main source of financial challenges after being diagnosed with cancer. A participant from Mexico reported her reliance on family for financial support after having to stop working due to cancer-related physical weakness.

*
I was already here in the US. I would help clean houses and at the same time I was separating from my partner so cleaning was my source of income. Well I stopped working because I knew that after my chemotherapy I would start to feel sick…My mom supported me in many ways, she would give me $30 to $40 weekly, food, or anything else that I would need...* (P39)

Family members also experienced employment disruption during their caregiving, potentially reducing the income. Various informal groups, such as church and family members, were able to offer support (e.g., nutritional supplements).

*
Well financially (impacted)… Because my husband is the only one who works. Sometimes he needs to take days off work so he can take care of me…You have to limit your spending as much as you can. Sometimes my family supports me financially. Having cancer comes with a lot of cost, I need a lot of vitamins to stay strong. My church has also helped me a lot. Last week they sent me a box of the vitamins that I need. Those are not cheap treatments, each of them cost about $120. They also said they were going to keep sending me more.* (P32)

## 4. Discussion

This study delineated challenges that Hispanic BC patients encountered regarding their cancer care throughout the COVID-19 pandemic. The array of COVID challenges in our study is similar to those in other cancer studies; however, unique challenges and their implications related to geographic and cultural aspects were at the forefront. Participants’ perceived concerns were reported in terms of challenges for timely care as well as psychosocial and financial well-being. 

The majority of our participants did not report the same extent in the disruption of cancer treatments (e.g., chemotherapy, radiation therapy) but focused on challenges in making appointments for diagnostic and testing purposes. Delays in cancer treatment may differ by the types of treatments. Diagnostic imagining and lab testing were the most commonly described treatments that were delayed during COVID [22]. Scholars reported that delays or disruption in cancer care during COVID were largely due to limited service availability [17].

The fear of becoming infected with COVID and its adverse impacts on cancer treatments (e.g., delayed treatment) were prevalent, similar to the findings from other studies [7,18,19,23,24]. While the general fear of a COVID infection is common, disproportionate hospital admissions and mortality rates among racial/ethnic minority patients including Hispanics were concerning. In the state of California, Hispanic deaths increased 31% during the first seven months of the pandemic [25]. Our participants’ direct and indirect experiences in the loss of multiple family members and friends perhaps increased threats to their own safety and fear of potential mortality. 

In line with other studies [6,7,26,27,28], our study found social isolation, especially from the required lockdown, was a major challenge and contributed to exacerbating participants’ sense of isolation and distress. The study participants who live in a border town of the US but had family members in Mexico experienced a heightened sense of isolation due to the border closure. However, a recent study with Mexican Americans living by the US–Mexico border found no impact of the COVID pandemic on family relationships [29]. Perhaps this was alleviated to some extent with the ability to maintain some familial contact via phone or videochat. Our study participants reported employing these lines of communication, but they were perceived as inadequate and limited in the ability to fulfill the needed affection and emotional support. Furthermore, physical separation imposed by international policy on the US–Mexico border closure might have imposed additional challenges for navigating BC treatment alone for some participants. 

The visitor ban in health care facilities imposed early in the COVID pandemic was particularly challenging for the participants who relied on their family for emotional and logistical support during their cancer care. Our participants found going to clinics alone quite distressing, as supported by other studies [7,24,30]. Family caregivers are an important source of health information and influence patients’ health care and medical decision making [31]. The Hispanic cultural value of *familismo* is a central source of emotional and instrumental support and plays an important role in one’s psychological well-being [29]. The absence of family and friends during health visits can negatively impact the participants’ need for support (e.g., translation) and should not be overlooked. This is more imperative for our participants, as the majority spoke Spanish as their primary language. Health care professionals’ facilitating language-concordant services or readily available translation services in Spanish would be crucial to help the patients’ information processing and, consequently, potentially reduce patients’ anxiety due to a lack of clear understanding. 

In addition, suspension or cancellation of non-essential cancer programs (i.e., support groups) during the pandemic minimized the availability for those who seek this type of social support. Scholars have suggested facilitating new ways of connecting among cancer patients during the COVID era. For example, web-based support groups (e.g., peer support, mindfulness-based interventions) or cancer online forums help decrease cancer patients’ psychological distress [32,33,34]. 

While these online-based resources can be optimal for providing a supportive environment, digital disenfranchisement is greater among those who are a racial/ethnic minority, have a lower educational status, and are of a low socioeconomic status [35,36,37,38]. For example, Black and Hispanic adults compared to White adults are less likely to have a desktop or laptop computer and home broadband [37]. Similarly, about 34% of those who have an annual household income less than $30,000 experienced difficulties in paying for high-speed internet services [38]. Hence, it will be important to assess patients’ accessibility and ability to use digital technology and develop a best practice to leverage telehealth among underserved populations [39]. Providing patient-level training, ways to engage others (e.g., family) when assisting patients, and technical support are areas needed to ensure the equity in telehealth services [40]. 

Lastly, another identified challenge of participants attributed to COVID was financial hardship. The COVID pandemic exacerbated problems with maintaining employment. A previous study [5] found that almost 22% of BC patients had decreased income post-COVID. Those who lost jobs struggled and employed various coping methods, such as relying on family financial support and cutting their own expenses. Financial distress can negatively impact the patients’ physical and mental health outcomes and cancer survivorship [41], highlighting the importance for health care facilities to screen for individuals’ financial concerns and needs [42]. In addition, scholars emphasized the need for a multi-level, systematic approach to include financial navigation assistance, including filling applications for financial services, connecting to community resources, and providing financial counseling [41]. 

### Study Limitations and Suggestions

There were some study limitations to consider. The participants were recruited from one study site in a border city area. Hispanic BC patients in the US–Mexico border region may be disproportionally affected by COVID through their unique challenges to access and utilize cancer care during the pandemic. Hence, future studies including cancer patients from other geographically diverse sites (e.g., rural regions) may provide more comprehensive information. Another limitation of our study is that the perspectives of health care providers and family members were not included. Although our study focuses on patients’ experiences in accessing cancer care, health care providers and family members are usually closely involved with patients. Eliciting their experiences in patient care and challenges encountered during the COVID pandemic may provide additional insight and solutions to address these challenges. Lastly, future studies assessing challenges to cancer care using validated measures and conducting a quantitative study can provide valuable data about its extent and scope, thus leading to insight in designing and developing effective interventions to address the participants’ needs in cancer care. 

## 5. Conclusions 

This study presents multiple levels of COVID-related challenges in cancer care ranging across medical, psychosocial, and financial issues among Hispanic BC patients from a border region. While COVID impacts on cancer care are challenging across the general population, this is particularly concerning for low-income Hispanic BC patients living by the US–Mexico border. They are subjected to the unique circumstances of geographic location and border policy between the two countries. Understanding the complexity of challenges faced by underserved minority communities is critical. Results of this study could increase health care professionals’ awareness about these potential areas of concerns. Mobilization and implementation of patient-centered approaches can improve patients’ physical and mental well-being.

## Figures and Tables

**Table 1:** Participants’ sociodemographic characteristics and cancer-related information (N = 27).

Variable	N (%)/M (SD)
**Age (years)**	54.4 (11.6)
**Level of education**	
Less than high school	5 (18.5%)
High school/GED	12 (44.4%)
Some college	6 (22.2%)
Bachelor’s degree	3 (11.1%)
Graduate degree	1 (3.7%)
**Annual household income**	
USD 30,000 or less	16 (59.3%)
USD 30,001 to USD 60,000	6 (22.2%)
Refused	5 (18.5%)
**Language of interview**	
Spanish	24 (88.9%)
English	3 (11.1%)
**Year of breast cancer diagnosis**	
Less than a year	15 (55.6%)
Between 1 year and 2 years	7 (25.9%)
Between 2 years and 3 years	2 (7.4%)
Between 3 years and 4 years	1 (3.7%)
Over 4 years	2 (7.4%)
**Stage of breast cancer**	
Stage 0	1 (3.7%)
Stage 1	4 (14.8%)
Stage 2	11 (40.7%)
Stage 3	2 (7.4%)
Stage 4	3 (11.1%)
Unsure/do not know	6 (22.2%)
**Degree of COVID-related impact on cancer care**	
Not at all	17 (63.0%)
Slightly	1 (3.7%)
Somewhat	5 (18.5%)
Extremely	4 (14.8%)

## Data Availability

The datasets generated and analyzed during this study are available from the corresponding author on reasonable request.

## References

[1] Rollston R., Galea S. (2020). COVID-19 and the Social Determinants of Health. Am. J. Health Promot..

[2] Daly M., Robinson E. (2021). Psychological Distress and Adaptation to the COVID-19 Crisis in the United States. J. Psychiatr. Res..

[3] Momenimovahed Z., Salehiniya H., Hadavandsiri F., Allahqoli L., Günther V., Alkatout I. (2021). Psychological Distress among Cancer Patients during COVID-19 Pandemic in the World: A Systematic Review. Front. Psychol..

[4] Caston N.E., Lawhon V.M., Smith K.L., Gallagher K., Angove R., Anderson E., Balch A., Azuero A., Huang C.-H.S., Rocque G.B. (2021). Examining the association among fear of COVID-19, psychological distress, and delays in cancer care. Cancer Med..

[5] Ludwigson A., Huynh V., Myers S., Hampanda K., Christian N., Ahrendt G., Romandetti K., Tevis S. (2022). Patient Perceptions of Changes in Breast Cancer Care and Well-Being during COVID-19: A Mixed Methods Study. Ann. Surg. Oncol..

[6] Rentscher K.E., Zhou X., Small B.J., Cohen H.J., Dilawari A.A., Patel S.K., Bethea T.N., van Dyk K.M., Nakamura Z.M., Ahn J. (2021). Loneliness and mental health during the COVID-19 pandemic in older breast cancer survivors and noncancer controls. Cancer.

[7] Savard J., Jobin-Théberge A., Massicotte V., Banville C. (2021). How did women with breast cancer experience the first wave of the COVID-19 pandemic? A qualitative study. Support. Care Cancer.

[8] Centers for Disease Cotnrol and Prevention (2022). Breast Cancer Statistics. https://www.cdc.gov/cancer/breast/statistics/index.htm.

[9] American Cancer Soceity Cancer Facts & Figures for Hisapnics/Latinos 2018–2020. https://www.cancer.org/content/dam/cancer-org/research/cancer-facts-and-statistics/cancer-facts-and-figures-for-hispanics-and-latinos/cancer-facts-and-figures-for-hispanics-and-latinos-2018-2020.pdf.

[10] Mullangi S., Aviki E.M., Chen Y., Robson M., Hershman D.L. (2022). Factors Associated with Cancer Treatment Delay Among Patients Diagnosed With COVID-19. JAMA Netw Open.

[11] Newman L., Fejerman L., Pal T., Mema E., McGinty G., Cheng A., Levy M., Momoh A., Troester M., Schneider B. (2021). Breast Cancer Disparities Through the Lens of the COVID-19 Pandemic. Curr. Breast Cancer Rep..

[12] Patel M.I., Ferguson J.M., Castro E., Pereira-Estremera C.D., Armaiz-Peña G.N., Duron Y., Hlubocky F., Infantado A., Nuqui B., Julian D. (2022). Racial and Ethnic Disparities in Cancer Care during the COVID-19 Pandemic. JAMA Netw. Open.

[13] Ko E., Cardenas V., Zúñiga M.L., Woodruff S.I., Rodriguez V., Palomino H. (2021). Challenges for Latina Breast Cancer Patient Survivorship Care in a Rural US-Mexico Border Region. Int. J. Environ. Res. Public Health.

[14] Ko E., Beloshapko A.V., Zúñiga M.L., Palomino H., Peacher D., Watson M. (2020). Binational cancer patient experiences and cancer coping in a rural US-Mexico border region. J. Psychosoc. Oncol..

[15] SANDAG: Binational. https://www.sandag.org/projects-and-programs/borders-and-interregional-collaboration/binational.

[16] Glass L.T., Schlachta C.M., Hawel J.D., Elnahas A.I., Alkhamesi N.A. (2022). Cross-border healthcare: A review and applicability to North America during COVID-19. Health Policy OPEN.

[17] Riera R., Bagattini Â.M., Pacheco R.L., Pachito D.V., Roitberg F., Ilbawi A. (2021). Delays and Disruptions in Cancer Health Care due to COVID-19 Pandemic: Systematic Review. JCO Glob. Oncol..

[18] Colomer-Lahiguera S., Ribi K., Dunnack H.J., Cooley M.E., Hammer M.J., Miaskowski C., Eicher M. (2021). Experiences of people affected by cancer during the outbreak of the COVID-19 pandemic: An exploratory qualitative analysis of public online forums. Support. Care Cancer.

[19] Leach C.R., Kirkland E.G., Masters M., Sloan K., Rees-Punia E., Patel A.V., Watson L. (2021). Cancer survivor worries about treatment disruption and detrimental health outcomes due to the COVID-19 pandemic. J. Psychosoc. Oncol..

[20] Phillippi J., Lauderdale J. (2017). A Guide to Field Notes for Qualitative Research: Context and Conversation. Qual. Health Res..

[21] Braun V., Clarke V. (2006). Using thematic analysis in psychology. Qual. Res. Psychol..

[22] Papautsky E.L., Hamlish T. (2020). Patient-reported treatment delays in breast cancer care during the COVID-19 pandemic. Breast Cancer Res. Treat..

[23] Seven M., Bagcivan G., Pasalak S.I., Oz G., Aydin Y., Selcukbiricik F. (2021). Experiences of breast cancer survivors during the COVID-19 pandemic: A qualitative study. Support. Care Cancer.

[24] Dieperink K.B., Ikander T., Appiah S., Tolstrup L.K. (2021). The cost of living with cancer during the second wave of COVID-19: A mixed methods study of Danish cancer patients’ perspectives. Eur. J. Oncol. Nurs..

[25] Riley A.R., Chen Y.-H., Matthay E.C., Glymour M.M., Torres J.M., Fernandez A., Bibbins-Domingo K. (2021). Excess mortality among Latino people in California during the COVID-19 pandemic. SSM Popul. Health.

[26] Jammu A.S., Chasen M.R., Lofters A.K., Bhargava R. (2021). Systematic rapid living review of the impact of the COVID-19 pandemic on cancer survivors: Update to August 27, 2020. Support. Care Cancer.

[27] Ayubi E., Bashirian S., Khazaei S. (2021). Depression and Anxiety Among Patients with Cancer During COVID-19 Pandemic: A Systematic Review and Meta-Analysis. J. Gastrointest. Cancer.

[28] Drury A., Eicher M., Dowling M. (2021). Experiences of cancer care during COVID-19: Phase 1 results of a longitudinal qualitative study. Int. J. Nurs. Stud. Adv..

[29] Volpert-Esmond H.I., Marquez E.D., Camacho A.A. (2022). Family relationships and familism among Mexican Americans on the US-Mexico border during the COVID-19 pandemic. Cultur. Divers. Ethnic Minor. Psychol..

[30] Moran H.K., Brooks J.V., Spoozak L. (2020). Undergoing active treatment for gynecologic cancer during COVID-19: A qualitative study of the impact on healthcare and social support. Gynecol. Oncol. Rep..

[31] Bevan J.L., Pecchioni L.L. (2008). Understanding the impact of family caregiver cancer literacy on patient health outcomes. Patient Educ. Couns..

[32] Kang C., Sun S., Yang Z., Fan X., Yuan J., Xu L., Wei Y., Tong H., Yang J. (2021). The Psychological Effect of Internet-Based Mindfulness-Based Stress Reduction on the Survivors of Breast Cancer during the COVID-19. Front. Psychiatry.

[33] Gallagher S., Bennett K.M., Roper L. (2021). Loneliness and depression in patients with cancer during COVID-19. J. Psychosoc. Oncol..

[34] Sillence E. (2013). Giving and Receiving Peer Advice in an Online Breast Cancer Support Group. Cyberpsychol. Behav. Soc. Netw..

[35] Early J., Hernandez A. (2021). Digital Disenfranchisement and COVID-19: Broadband Internet Access as a Social Determinant of Health. Health Promot. Pract..

[36] Eruchalu C.N., Pichardo M.S., Bharadwaj M., Rodriguez C.B., Rodriguez J.A., Bergmark R.W., Bates D.W., Ortega G. (2021). The Expanding Digital Divide: Digital Health Access Inequities during the COVID-19 Pandemic in New York City. J. Urban Health.

[37] Atske S., Perrin A. Home Broadband Adoption, Computer Ownership Vary by Race, Ethnicity in the US. https://www.pewresearch.org/fact-tank/2021/07/16/home-broadband-adoption-computer-ownership-vary-by-race-ethnicity-in-the-u-s/.

[38] Mcclain C. 34% of Lower-Income Home Broadband Users Have Had Trouble Paying for Their Service amid COVID-19. https://www.pewresearch.org/fact-tank/2021/06/03/34-of-lower-income-home-broadband-users-have-had-trouble-paying-for-their-service-amid-covid-19/.

[39] Bayard S., Fasano G., Gillot T., Bratton B., Ibala R., Taylor Fortson K., Newman L. (2022). Breast Cancer Disparities and the Digital Divide. Curr. Breast Cancer Rep..

[40] Dixit N., Van Sebille Y., Crawford G.B., Ginex P.K., Ortega P.F., Chan R.J. (2022). Disparities in telehealth use: How should the supportive care community respond?. Support. Care Cancer.

[41] Thom B., Benedict C., Friedman D.N., Watson S.E., Zeitler M.S., Chino F. (2021). Economic distress, financial toxicity and medical cost-coping in young adult cancer survivors during the COVID-19 pandemic: Findings from an online sample. Cancer.

[42] Edward J., Petermann V.M., Eberth J.M., Zahnd W.E., Vanderpool R.C., Askelson N., Rohweder C.L., Gonzalez S.K., Stradtman L.R., Ko L.K. (2022). Interventions to address cancer-related financial toxicity: Recommendations from the field. J. Rural Health.

